# Notes on Medical Matters and Medical Men in Paris

**Published:** 1847-11

**Authors:** David W. Yandell

**Affiliations:** Louisville, Ky.; Paris


### WESTERN JOURNAL

OF

### MEDICINE AND SURGERY.

NOVEMBER, 1847.

### Art. I.—*Notes on Medical Matters and Medical Men in Paris.* By David W. Yandell, M.D., of Louisville, Ky.

I begin this letter with sketches of some of the most distinguished Physicians of this metropolis; how it will be concluded will depend upon the topics which may emerge in the next few days. It is not always in the power of a student, even in Paris, to impart that variety to his letters which "the mind of desultory man" enjoys; but, as I take the aim of your readers to be something higher than the simple gratification of taste, I am not without hope that they find an interest in my merely professional details. The mingling of the *utile* with the *dulce* is a feat reserved for the favored few to execute. Most mortals must be content with achieving the first, and happy should they be to feel that they have been useful.

*M. Chomel*, professor of clinical medicine at the Hôtel Dieu, is in his sixtieth year, though I think if it were not for a stoop in his shoulders he would not appear so old. The expression of his face is pleasant and determined. His head is round, and not badly proportioned; his eyes are black, shaded by handsome eyebrows; his nose turned up, his mouth small and round, his hair thick and gray. In manner he is considerate to his pupils, kind and conciliatory to his patients. Like the majority of French lecturers, he speaks fluently, rapidly and distinctly. Making usually but few clinical remarks at the bedside, his service cannot be considered interesting, unless his lectures—which during the winter he delivers three times a week in one of the amphitheatres of the hospital—be considered as forming a part of it, when it is at least not wholly devoid of interest, if lacking in that spirit which makes the service of some of his colleagues so attractive.

*M. Rostan*, also professor of clinical medicine at the Hôtel Dieu, I should say was near the same age as M. Chomel, though he is a younger looking man, of medium height, round body, broad, short, open and agreeable face, good head, medium forehead, thin, grayish hair wanting on top, hazel eyes, small mouth, and common nose. This portrait may not strike you as being very like the engravings that we see of the great American philosopher; but I never was more forcibly impressed with the resemblance between any two faces than when, coming one morning from the Hôtel Dieu where I had seen M. Rostan, I saw an engraving of Dr. Franklin. Rostan is a pleasing and occasionally an animated speaker, clear in his reasoning, and careful in his deductions, and always exhibiting in his remarks, both at the bedside and in the lecture room, the warmest desire to communicate sound, valuable and practical knowledge to the troop of students, native and foreign, who attend his clinics.

*M. Andral* is a very ordinary looking man, fifty years of age, five feet eight or ten inches in height. His hair is grayish, thin on the crown of a medium sized head, long behind and in front. With a stoop in his carriage, a slim and not very well formed person, a large and long nose, and thick lips, no one would ever point him out as the professor of pathology and general therapeutics in the *Ecole de Médicine.* His service at *La Charité* is attended by few, in fact by no students, because he does not seem to care about imparting instruction at the bedside. As a lecturer I spoke of him in a former letter.

*M. Bouillaud*, professor of clinical medicine at La Charité, in many respects is Andral's opposite. His carriage is erect, his head unusually fine, his forehead broad, round, high and full, his eyes dark and animated, overhung by black, thick eyebrows; his hair thin, long, and black, sprinkled here and there with gray; for he, too, is fifty years old. His face is thin and his cheek bones high, his nose large and one of the most expressive features of his countenance. Intellectuality and intensity are the predominant and indelible characteristics of the man. In his bearing towards his patients he is unnecessarily severe and abrupt, and towards his students touchy and ill-tempered. He lectures with great earnestness, fire and grace. His sentences are now round, beautifully turned, ringing and compact, and again, as he becomes engaged in the discussion of his favorite doctrines, or even when presenting some important question, his thoughts flowing as rapidly as lightning, and growing more earnest as he proceeds, they become nervous, and sometimes abrupt, and almost jerking.

*M. Louis*, one of the physicians at the Hôtel Dieu, is a striking looking, finely formed man, standing six feet in his socks, sixty years of age, with a fair head, high forehead, thin, short, brown hair, broad, thick whiskers, straight, handsome nose, crossed by a pair of spectacles, well shaped mouth, round chin, and blue eyes. He struck me as being a fine looking, sour-tempered man, particularly unkind to his patients, and not especially pleasant to the three or four pupils who follow him.

Of *M. Rayer*, author of an able work on cutaneous diseases, I have already spoken.

*M. Cruveilhier* is a round, pleasing, amiable looking man, fifty-six or fifty-seven years old, five feet seven or eight inches in height, with regular, handsome features, eyes dark and large, mouth expressive of amiability, hair gray, and small whiskers of corresponding color. His manner to his patients is kind.

As a lecturer he ranks low, probably lower than any other professor in the School of Medicine. His voice is sadly against him, being weak and without either volume or music. He speaks without animation, in a singsong, and dwells long upon what is frequently least important in his subject, often seeming not to seize—as he certainly in many instances fails in developing—the cardinal points of his matter. Very few students attend his lectures; and when they have been, as I may say, spoiled by listening to such speakers as Denonvilliers, Dumas, Trousseau, and others of the same order, I only wonder that any at all have the patience to *endure* him through an hour. I say endure him, because one must have good ears, keep them well open, and sit close to him, in order to hear the fifth part of what he says.

*M. Trousseau* is an elegant looking, and very handsome man, of fair complexion, long, black hair, which he combs directly back from his face, long Bourbon nose, blue eyes somewhat sunk in his head, which is of medium size. He wears broad, thick, half-whiskers. He is an orator. Clear, earnest and graceful, with a fine voice, person and eye, he at first commands the attention of his auditory, and then fixes it by the attractive manner in which he presents the well digested matter of his numerously attended and instructive lectures. M. Trousseau is not yet quite forty-seven years of age, and is, as you know, professor of therapeutics and materia medica in the School of Medicine.

*M. Piorry*, professor of internal pathology in the School of Medicine, is about fifty-three years old. He is a tall, round-shouldered man, with a long face, long nose, wide mouth, thin lips, small black eyes, and immense black whiskers. He speaks well, that is to say, as well as a man without grace of manner or person, or flexibility of voice, can speak. His style is earnest, and could he throw a little grace into his gesticulations, and give the least possible softness to his voice, he would please much more than he does. The expression of his face, like the tones of his voice, is *hard*, and not intellectual, but firm, unbending and determined—in a word, indicative of the character of the man. Laborious and persevering, no task and no number of defeats deters him from the pursuit of his hopes. He said, while a young and unknown physician, "I will one day fill a chair in the School of Medicine." Five unsuccessful *concours*, so far from disheartening him, seemed to inspire him with new and greater determination, and at the sixth, triumphing over every obstacle, he demonstrated in too unmistakeable terms his capacity, and was raised to the chair which he now occupies. His eye is sharp and penetrating, and with his head slightly sunk between his broad shoulders, his huge whiskers, and his stony voice, he strikes one, after all—at least he did me—as being by nature better fitted for a commander of infantry than a teacher of medicine.

*M. Dubois*, professor in the faculty, (*Clinique d'Accouchement à l'Hôpital de la Faculté*,) is about the age of M. Piorry. He is not so tall, and is the mildest, most amiable, benevolent and harmless looking man imaginable. His complexion is fresh; his hair, wanting on the crown, is brown, and thin on the sides; his nose is long, his chin long and round, and his eyes blue. There is a singular depression about the center of his head, seeming as if produced by some sharp-edged heavy body, which had pressed gradually upon the middle of the parietal bones and raised up the frontal and posterior portion of the cranium. As a lecturer, he is pleasant, popular and practical.

These are unfinished and defective portraits—copied from my note-book—of some of the principal Parisian physicians. In all cases I have given my opinions candidly and my impressions fully. I have spoken and will only speak of the different gentlemen as they appeared to me in the wards and amphitheatres of the hospitals, and in the halls of the School of Medicine. And now, after having, with probably as unprejudiced a mind as one could well possess, followed in their visits and attended some of the lectures of the greater part of the men to whom I have alluded, my opinion is, that Bouillaud, of La Charité, is the greatest man of them all; and if to his other qualities he joined the kindliness of manner to the patients entrusted to his care possessed by Dr. Norris, of Philadelphia, I should say that he was my choice of all the physicians I have ever seen. I do not here speak of his doctrines, as I do not intend speaking of those of any of the others; but I wish to be understood as alluding especially to his powers as a diagnostician.

Chomel seems to me to belong to the bygone generation. He is at best but an ordinary man, and owes the professional position which he occupies as much to his kindliness of manner to his patients and his pupils, aided by other circumstances, as to any particular strength of mind or fund of knowledge.

Rostan, as a teacher at the bedside, is without an equal in Paris; but he has occasionally wandered from the austere and rugged paths of science, and worshiped at other than her altars, and, as is inevitably the consequence, except in men of the highest order of mind—and in some cases even with them—although endowed with a fair share of talents, and of very popular manners, he has halted this side the great goal, and will never be canonized.

Andral belongs to no common order of men; but there is ever an uncertainty in his mind as to his diagnosis—a laboring after something which he does not reach—a timidity in the formation of his opinions, and a hesitancy in their expression, which deters one from forming that estimate of him which we might otherwise be disposed to do.

Cruveilhier, while considerate to his patients, is a better anatomist than physician.

Rayer, with all his cumbrousness of person, is unquestionably the possessor of a clear, far-seeing, bold and discriminating mind. His wards usually abound in interesting cases, but from his making so few remarks his visits are comparatively profitless to the student.

Louis is not the great man in Paris that he is in the United States; but divesting the mind of the opinion entertained of him in either place, is he a great physician? I answer unhesitatingly in the negative, to this question. As we all have our own ideas of a good lecturer, so we have them of a great physician. To be a great physician, a man must not only be able to arrive at a diagnosis, but must be able to do so quickly; he must not only be able to ask questions of the patient which will put him upon the desired track, but he must do it and not confound the person with a long, tedious and useless catechising; and above all things else, his diagnosis made, and his prognosis given, the autopsy—if the disease terminate fatally—must support the one, and the result of the case confirm the other. Louis can make a diagnosis, but it is always after a long examination of the disease, and useless questions to the patient; and too often, as any one who has followed him in his visits can testify, has his diagnosis been contradicted at the autopsy, which at last, in most cases, is the only true and legitimate method of ascertaining the capacities of the physician, and the accuracy and value of his knowledge.

I look upon Andral as superior to Louis; Rayer as superior to Andral; and Bouillaud as superior to them all. There is a cast about the mind of Bouillaud which in our eyes is to be preferred to that of any of the others. Bold, vigorous, quick and acute, he is endowed with perceptive powers to an unusual extent. Original and well disciplined, he generalizes and classifies with rapidity and correctness; discriminating, he readily detects error, and, fearless, he independently exposes it. His powers of generalization, backed by a strong disposition to exercise them, have undoubtedly led him to advance principles which many of his brethren deem untenable. The quickness of his perception conveys him with astonishing celerity to the core of a subject, and he is vexed because others, less fortunate in this particular endowment, still traverse with slow and measured steps what he has passed at a single bound. Bouillaud's diagnosis made, he reveals it on the spot. Louis, as a general rule, although doubtless in the majority of cases he has arrived in his own mind at the diagnosis of the disease, does not avow it, and it is sometimes four or five days before he allows it to leak out. It would be folly to question Louis's knowledge of the physical signs of disease, but the want of powers of combination, which we suppose no unprejudiced man will claim for him, cripples him in his deductions, biases him in his opinions, creates distrust of them in his own mind, and inspires an unpleasant doubt of their correctness in the minds of those who follow him in his visits. Bouillaud, it may be said, is over-confident. The rapidity with which he diagnoses would at first lead one to this opinion; but then, if one will take the trouble to eliminate gradually what he has probably deduced on the instant, he will find in the majority of instances that Bouillaud has reason to be confident, because he has arrived at the truth. Bouillaud is irritable—he belongs to the *genus irritabile;* Louis is irritable, without being able to establish any claims to membership in the same class.

*Cerebral Affections.*—M. Rostan is in the habit, every year, of devoting a few clinical lectures to the special illustration of diseases of the brain, in which department of science, as you are aware, he has done more than any living physician. In one of his late lectures he examined the value of paralysis as a diagnostic sign. His remarks may be summed up as follows:

Paralysis is one of the most striking and unerring signs which can exist to mark the seat of cerebral lesions. It should be studied in its seat, extent, intensity, march, duration, and in all the different circumstances which may impart to it different characters. The seat of the paralysis indicates, in the first place, the part of the brain where we find the organic alteration; but much is required to be able, after that, to determine precisely in every case the extent of this alteration. If a circumscribed paralysis corresponds to a circumscribed cerebral alteration, it is possible that this paralysis, occupying both the arm and leg of the same side, may be produced by a simple lesion of small extent, situated upon the confines of those portions of the brain which preside over the movements of the two members, and act equally upon the one and the other. In the appreciation of the extent of the lesion, you must take into account also the intensity of the paralysis; but you must not think that in this respect the relations are mathematical; for an organic lesion of circumscribed extent may produce a very complete paralysis, as, on the other hand, an extensive lesion may give rise to a paralysis so slight, that one may even in some degree doubt whether there really exists any anatomical disorder or change. When I first discovered *ramollissement* of the brain, I feared that it would not be possible, during life, to distinguish this from other alterations of that organ—that, occupying the same situation, it would manifest itself by the same phenomena.

But the mode of invasion, the march of the disease, will not be the same as those, for example, of cerebral hemorrhage. They differ in effect; whence the necessity of bearing in mind the march of the affection. If the paralysis is sudden and without precursory phenomena, it is extravasation of blood—cerebral hemorrhage—that you should suspect in the great majority of cases, remembering at all times that there are exceptions to the rule. Thus in certain cases of *ramollissement*, while the lesion is not very extensive, it may produce no sensible trouble; but the day comes when its volume is such that suddenly the phenomena appear with fearful rapidity. This is the drop of water which overruns the vase. The analogy is true. As there are *ramollissements* without precursory phenomena, so these phenomena may occasionally precede the congestion or cerebral hemorrhage.

After examination of the seat and the extent and precursory phenomena, you must look at the march of the affection. A paralysis declares itself, and augments day by day; suddenly there may supervene a diminution of the symptoms, a passing amelioration, and this is a circumstance which may lead you into error; for paralysis, even when increasing, has not always a regular march. Under the influence of a sanguine emission, the compression of the pulp may momentarily cease, and consequently the paralysis diminish. This now explains how there may be greater or less alteration in the intensity of paralysis. The paralysis which succeeds cerebral hemorrhage may disappear or persist, according to the manner in which the effusion is made. When the blood has simply separated the cerebral fibres, you can easily understand that in the proportion and measure of its absorption the phenomena of paralysis will disappear; when it has destroyed the cerebral fibres. then the power of motion is wholly lost, and it is in vain that you employ electricity, nux vomica, blisters, etc.

I remember two curious cases of paralysis analogous to some of which you have perhaps read. One was a subject who had been paralytic for many years; he could not walk; his house took fire; he sprang from his bed, walked alone to escape being burned, and was cured. A hemiplegic woman was received at Salpêtrière. Some of her acquaintances put her in communication with a miracle-worker, Prince Hoenlohë. Prayers were said for nine days; at the end of the time predicted by the prince, the patient was cured, having recovered her movements. This case made so much noise that the governors of the hospitals believed it their duty to institute an inquiry, and they asked me my opinion. My reply was simple, and destroyed all the marvelousness of the miracle. In the first place, it is possible that a moral cause might act upon the brain like the blow of a club upon the shoulder, and that there may not have been a material destruction of the encephalic substance. In other cases there have been hemorrhages, effusion between the cerebral fibres; the effusion is absorbed, and motion does not return, simply because the patient has lost the habit of exercising his limb. Let a cause, it makes no difference what, compel this movement, and the patient is cured. With this woman two things were possible: either that she was in this last condition, and the persuasion that she would be cured had actually given her this movement; or, what is also supposable, she had simulated paralysis in order to enter the hospital, and not finding it quite as agreeable a place as she supposed it would be, became weary of her situation and resolved to change it.

From the nature of the lesion by which it has been produced, it follows that paralysis terminates by recovery, or increases, or remains stationary, and that the treatment should be directed not against the paralysis itself, which is but a symptom, but against the lesion which has given rise to it.

*Etherization in Meningitis.*—M. Besseron, physician of the hospital of the mustapha, in Algeria, addressed a note to the Academy of Sciences upon the employment of etherization in the treatment of cerebro-spinal meningitis. This agent appears to have produced some good results in this affection. M. Besseron has observed that in patients thus treated, there is at first a little exaltation, soon followed by a sedation, of which the effects have been advantageous.

*Structure of the Brain.*—M. Pappenheim sent a paper to the Academy of Sciences upon the structure of the nervous centers. He has endeavored to penetrate the profound mechanism of the structure of the encephalon. The general conclusion of his memoir is, that the encephalon is composed of plates or lamellæ—an external, a middle and an internal plate. The nervous trunks arise only in the middle lamella. This disposition does not exist in all parts of the brain; when it does not exist, the nervous trunks arise from two lamellæ at once; but whenever there are three, they arise only from the middle.

*Luxation of the Elbow.*—There is an example of this luxation in our wards, said Professor Roux a short time since, which affords me an opportunity of offering you some remarks upon the nature of this lesion. The nature of the case was not recognised by the physician who was first called in. You have never known me, he remarked, especially at the bedside, accuse a professional brother of having committed an error; the blame, always out of place, will often be unmerited when it concerns a traumatic affection of the elbow. The articulation is studded with osseous projections which cannot be felt, or which may be confounded the one with another, because of swelling, particularly in women, and still more among children, when they are but slightly disclosed. Our best surgical treatises are, moreover, as regards the diagnosis of these luxations, indefinite and unsatisfactory. For example, they tell you that in the displacements backwards the forearm is always flexed, and that this flexion, with the projection of the olecranon behind, will constitute the two essential characters of the lesion. Now, very often the position of the limb is nearer that of extension than flexion, and in trusting to the pretended constancy of this sign you will be led into error of diagnosis. The displacement is overlooked, especially in children and women, where the osseous projections, as we have said, and the muscles are less clearly defined beneath a tumefaction which, although varying in degree in different cases, is always present. It requires sometimes long practice to avoid this mistake. I have seen ten or twelve dislocations of the elbow thus overlooked, or, to speak more plainly, misunderstood, and left for a long time without any attempts being made to reduce them. It is a fortunate circumstance that after many weeks, and even after many months, we may still very often succeed in reducing them. If we fail to do so, the motion of the joint is in a great degree lost, and the limb remains permanently extended just as if it were composed of one piece.

I will cite you to a case of success: I choose it among those that I have myself observed, because, apart from the pathological interest which attaches to it, there is something of romance connected with it. It was twelve years since, in a foreign country. I was at Milan, having just returned from Pavia, (where, I may remark in passing, I saw the head of Scarpa macerating, unhonored, among other anatomical specimens in a tub.) On my return from this excursion, I found some gendarmes awaiting me at my hotel. At that time recent troubles prompted stringent measures of precaution concerning travelers. For a moment I was a little alarmed. The commandant of the detachment advanced towards me, announcing as he approached that he came to consult me about his son. As you may imagine, I was instantly relieved. His son, who was in the Austrian cavalry, had about five months before fallen from his horse and dislocated his elbow. The nature of the lesion escaped the first physicians who were called in. Panezza, and the successor of Scarpa, to whom the patient afterwards presented himself, said they had seen the luxation too late, and made no attempt to reduce it. I examined the elbow; the displacement was evident; there was scarcely the least mobility between the bones. I told the father of the patient that I had often reduced ancient luxations, but none had been in a state similar to this; that I was desirous of attempting it, however, without warranting success; that at any rate there was no danger from prudently directed efforts. I employed a number of robust men in extension and counter-extension, and succeeded. I have seen since many similar examples of success.

Carried too far, these attempts at reduction may produce serious accidents. Unnatural adhesions may be formed, and I only wonder that we do not more frequently fail. I myself have never observed any other accidents here than the fracture of the olecranon; it occurred at the moment when the flexion of the forearm completed the reduction. There was no laceration of the integuments, nor any other unpleasant consequences; but the joint regained its mobility only very incompletely; extension remained limited, which, however, offered less inconvenience than if the limit of motion had been in the opposite direction.

Fractures of the elbow also present great difficulty of diagnosis. It is often hard to pronounce upon the seat, direction, and sometimes even upon the existence of a solution of continuity of the bone. This is easily understood: the least derangement in a machine—the breaking of one of its wheels, the bending of one of its teeth—and its movements are clogged; the apparatus stops. The same is the case with the articulations, and especially with those that are unequal, as of the elbow. We feel that it is not always an easy matter to perceive the spoke of the wheel which has suffered, and the exact alteration which it has experienced. We must be careful in the diagnosis and reserved in the prognosis, remembering that simple contusions of the elbow, especially among children, may lead to anchylosis. If in practice credit has sometimes been given us for cures in which nature has largely contributed to success; more often still have reverses been laid at our door for which the gravity of the lesion alone was responsible. One of my best pupils and one of our most distinguished provincial surgeons, M. Fleury, Jr., had given the most assiduous and enlightened care to a fracture of the elbow; pronation and extension unfortunately remained embarrassed, and it was impossible for me to persuade the patient that it was not the fault of the surgeon. You must not be too sensitive, but prepare yourselves for similar reproaches, which the most able have received before you.

*Sarcocele.*—We are going presently to operate, continued M. Roux, for a sarcocele; but before doing so, I wish to say a few words concerning the complication, or, more correctly, the coincidence of hydrocele with cancer of the testicle, for this dropsy may be the source of a singular error of diagnosis. The serous effusion may disguise the alteration of the gland; an optical illusion may induce the belief that the serosity is greater than it really is, and the solid tumor less voluminous. A thin layer of any limpid effusion gives a transparency out of proportion with the small quantity of the liquid, and diminishes to the same extent the mass of the testicular tumor. The transparency, which is the sign, *par excellence*, of hydrocele, may then be deceptive in masking, by the exaggeration of its own extent, a tumor or degeneration of the organ. This is a very curious effect of a small quantity of clear liquid uniformly distributed around an opaque mass. You may give yourselves an idea of it by looking at one of your hands held between you and a bright light; it appears of a general transparency, while nothing indicates the position of those parts impervious to the light, the phalanges and bones. In sarcohydrocele this illusion is extremely apt to mislead. I will relate to you the first example that I ever saw of this fallacious phenomenon. A surgeon of Orleans—one of my cherished pupils, M. Lévêque, whose thigh I was obliged to amputate, and whom science lost twelve years after—sent me the patient. The mother of the man having been successfully operated on by M. Boyer for a cancer of the breast, naturally had the desire, in which I participated, to have the advice of that able surgeon. M. Boyer examined him, and, deceived by the illusive appearances which I mentioned, decided upon the spot that it was nothing more than a hydrocele, expressing his astonishment that any one should have sent the patient all the way to Paris for so simple an affection. M. Boyer had only been struck by the transparency, the semiological value of which he had done a good deal towards establishing, and not by the antecedents, nor the weight of the tumor. As he was somewhat of a talker with his patients, he said to this one that he was very fortunate in having only a hydrocele, for sarcocele was almost always reproduced after the operation. Some days after, I was thoroughly convinced that it was a sarcocele, and so expressed myself. The patient, placed thus between two conflicting opinions, called a consultation, and the cancerous nature of the tumor was recognised. An exploring puncture gave issue to only three spoonsfull of serum. This small quantity of liquid had sufficed to produce this general transparency, by which my venerable master had allowed himself to be imposed upon. Castration was performed, and the patient is living to this day. By an extraordinary chance I operated during the same year for three sarcoceles, which have not reappeared. If relapse is to be feared in such cases, we should not exaggerate the danger.

In the patient who is the subject of this lecture, there is not the slightest uncertainty. The disease commenced fifteen or twenty months ago; a hydrocele would have attained a greater volume in the same length of time; the size being equal, it would be a little less heavy; the finger, in depressing the parts, arrives upon a solid mass which evidently forms almost the whole of the tumor. A puncture previous to operating moreover will dispel any uncertainty. The cord is healthy, and there is no appreciable lymphatic engorgement in the abdomen. This is, except a light effusion, sarcocele in all its simplicity. The operation will offer then the same character. In this there is but one peculiarity to watch—that is, the tendency of the mouths of the external pudic to retraction if they are not tied as soon as exposed; and if afterwards one is unable to ligate them, there will be great danger of secondary hemorrhage.

A puncture gave issue to a spoonful or two of serum. The patient was etherized with success. A vertical incision was made in the integuments, the tumor stripped, the cord isolated, seized and divided; a dry cloth was first applied upon the wound without cerate, then some rolls of lint, long compresses, and finally the inguinal bandage. The testicular substance was indurated and scirrhous, and the epididymis offered masses of tuberculous matter.

Thirty years ago, when surgeons did not fully appreciate the assistance that might be derived, in the diagnosis of hydrocele, from the transparency, the affection was very often mistaken and treated accordingly—that is to say, by castration. Pott, of England, and J. L. Petit both frankly confessed this fault, which I myself committed twice at Beaujon hospital in the commencement of my practice.

I remember another example of this mistake, of which a distinguished surgeon was the author, but fortunately failed being the victim. It was the surgeon himself who was affected with a scrotal tumor. He believed it to be a sarcocele, and, as you may suppose, it made a profound and disheartening impression upon him. What contributed to confirm him in the conviction that his tumor was of a cancerous nature, was the irregular manner of its growth, which had been, so to speak, by jerks, and the sharp pains that he felt or imagined he felt in it. He had diagnosed and operated for more than three hundred hydroceles, and he knew not how to recognise his own. He made up his mind and resigned himself to the operation. He desired that I should perform it. At first sight, before endeavoring to discover the transparency, and notwithstanding the weight of the tumor, I thought of a hydrocele. The transparency was perfect.

This transparency is an admirable sign, and I would that I knew who discovered it, in order that I might return him thanks for the important service he rendered to humanity. But you must not look upon this as an infallible sign and never deceptive. We have seen, on the contrary, in the case of hydrocele, that in becoming exaggerated by the limpidity of the effusion, it augments to the eye of the observer this effusion, at the expense of the solid part of the tumor, and may thus lead you to take a sarco-hydrocele for a hydrocele. You know, moreover, that the opaque nature of the liquid or the thickness of its coverings may render it completely null; so that its absence does not indicate more surely the absence of hydrocele than its existence does that of the same affection. But these exceptions are so rare, that transparency is one of the best elements of diagnosis possessed by the surgeon.

To return to our patient, I will complete in two words his history, in saying that I could not induce him to believe that he had a simple hydrocele until he saw the liquid escaping by the canula. I threw up the vinous injection, and he recovered and lived many years.

The case on which we are going to operate today in the same manner, is a type of the affection of which it presents all the characters very clearly expressed.

M. Roux, after making two vinous injections, as is his custom, proceeded to speak of

*Venereal Testicle.*—Among the consecutive symptoms of syphilis, one of the most remarkable, without doubt, is the venereal testicle. In the first place, it is one of those which, exceptionally to the rule, is developed in the neighborhood of the seat of the primitive symptoms. The ancients have left us but little concerning this affection, and what still offers the greatest difficulty today is the diagnosis. Nevertheless, we may sometimes recognise it at first sight, as we have done in the patient who is the subject of these reflections. In fact, the tumor has in general a very important exterior character, —which has been signalized by the English, and which I have noticed in my *Voyage à Londres*—which is its pear shape, with the base below, so that the tumor seems to encroach upon the cord or to be continued with it by a kind of stem or stalk. If this form is not constant, it is very frequent. It is as rare that tubercles and hypertrophy of the testicle affect the two sides, as it is common for syphilitic engorgement to do so. It is equally almost without example that sarcocele is observed simultaneously or successively on the right and left sides. It is the same in cancer of the breast in the female, and I have not seen this manifest itself on both sides more than five or six times. It is a very curious thing that this degeneration does not attack both the organs except in the case of diathesis. For the venereal and cancerous testicle there is an excellent touchstone, which is mercurial frictions; they rapidly cure the one and aggravate the other.

Fifteen years ago, a man from the country presented himself to me with a very voluminous testicle. I diagnosed a sarcocele, and declared the operation to be urgent. Before deciding to submit to it, the patient went to get the advice of another surgeon, and committed the fault of telling him my opinion. This confrere cheered up the patient by telling him that he only had a venereal testicle, which he would promise to cure by mercurial frictions. He tried them; the pain as well as the volume of the tumor was augmented, and in the course of six weeks the sarcocele had become but too incontestible, and it was necessary to perform the operation, the chances of success in which had greatly diminished.

As to our patient, he had three months ago a gonorrhea and chancres, which were badly treated, especially the chancres; and this is the worst of all the venereal symptoms—that which exposes most to consecutive symptoms. The tumor is pyriform, etc.; in a word, the sympathetic nature of the engorgement of the testicle is manifest, and in a little time the mercurial frictions will do justice to it. Professor Roux then went on to speak of

*Superficial Gangrene.*—Four cases of superficial gangrene at the surface of surgical wounds have occurred in our wards within a short time past, which I may as well recall to your minds by a few very brief remarks.

1. A woman whose breast had been removed. The wound was in full suppuration, when an eschar formed at the surface, to be detached, in almost a single piece, at the end of four or five days, from which time recovery was in nowise retarded.

2. Same accident in another woman who had undergone a similar operation. This one died.

3. A young man had a phlegmon at the groin, following an adenitis, which I opened with the bistoury. Same complication; application of the actual cautery; recovery.

4. The last case is that of a man who had been operated upon for a voluminous sarcocele. After a light chill, the wound assumed a black tint, as if an extremely thin eschar covered its surface. The patient grew no feebler, but the condition of the wound did not amend notwithstanding the treatment pursued. Among the applications to which I had recourse, I will cite the concentrated solution of nitrate of silver, the employment of which was suggested by an ancient *interne* of this hospital, who now practices with distinction in one of the provinces. He recalled to me in his letter a circumstance that I had forgotten, and which you will not regret knowing. In consequence of the removal of one of the tonsils, this physician, then a student, had such abundant and repeated hemorrhages that they inspired serious fears, and demanded the application of ice, and finally of the actual cautery. This is a very rare case; it would perhaps be impossible for me to cite a second example in my practice. Scarcely escaped from the hemorrhage, his life was again endangered, this time by the very means that had saved it, namely, the refrigerants. A pneumonia was developed, which seemed for a time about to carry him off.

This physician writes me that he believes he has found the means of preventing phlebitis and purulent infection from wounds, in cauterizing with the concentrated solution of nitrate of silver; that this caustic, crisping the orifice of the vessels, opposes the absorption of pus. Whatever may be the theory, this new therapeutic agent was presented as resting upon three cases of complete success; and I have tried it, but must declare that, far from being able to praise it, I am rather induced to think that it augments the gangrenous state of the wound. True, it may have failed in this case, and nevertheless fulfil the indications claimed for it.

Be this as it may, these four patients were etherized. I ask if this superficial gangrene of the wounds, which did not have,—at least, did not appear to me to have—the aspect of hospital gangrene, was altogether independent of the use of the ether? I had made up my mind as regards this question, but still it seemed to merit being considered. This is an accident so exceptional, that it is difficult to admit in its development the intervention of this new agent, now so universally employed, and which moreover will retain its place in the practice of surgery. In extenuation or exculpation of the ether, I may mention to you a singular case of gangrene manifesting itself, without any appreciable cause, in the groin, after an operation for strangulated hernia performed by M. Blandin. Etherization had not been employed, and yet gangrene of the wound arose and progressed to considerable extent. So far. this gangrene, in operations under the influence of ether, has only been observed in the Hotel Dieu.

On the 28th of June, M. Andral read to the Academy of Sciences the following paper

*On the State of the Blood in a Case of Scurvy.*—While Messrs. Becquerel and Rodier were collecting the materials for a memoir which they communicated to the Academy, on the state of the blood in scurvy, I observed at La Charité, in April last, an analogous fact, which also tends to establish, contrary to what has been clearly proved, in more than one case, that scurvy may manifest its existence, by the best marked and gravest symptoms, without the blood showing any alteration, either as to its aspect or the proportion of its fibrine. The case to which I am about to call the attention of the Academy relates to a man sixty-one years of age, who for many years has been progressively growing feebler, and who at the time of his entrance into the hospital present ed all the symptoms of a scurvy already far advanced. Numerous petechiæ and large ecchymoses were scattered over the members and trunk. Blood flowed almost continually from the nostrils, and it was easily pressed from the gums. The patient, of a waxen yellow hue, was in a state of extreme debility; upon the slightest movement, his respiration became much embarrassed, his heart beat violently, he felt excessively giddy, and was menaced with syncope; he could scarcely take a few steps without being upon the point of losing his consciousness. He had extreme repugnance to aliments, and the little food that he was able to take was very painfully digested. The pulse was ordinarily at 68. One day the patient was found with a hot skin, frequent pulse, dry tongue, and an oppression such that he seemed to be threatened with death from suffocation; he had an almost incessant cough. Auscultation and percussion could be but imperfectly practiced; a strong congestion of the lungs and bronchi was developed, and, seeing the manifest feeble reaction which existed, there was a chance of relieving the patient by drawing from him a little blood. A small bleeding was accordingly practiced, and was followed by a momentary amendment of the symptoms; the respiration especially becoming immediately less embarrassed.

This blood I examined. I was prepared to find it diffluent and dissolved; but, to my great astonishment, it was not so. It was in fact constituted by a small clot, dense and resisting as the clot of the phlegmasiæ; this clot was covered by a perfectly characteristic coat; it was besides of a very small volume, and remained as if suspended in the midst of a great quantity of serum. In its aspect, this blood in no respect resembled that ordinarily taken from scorbutics. It had, on the contrary, the aspect that I have often found in the blood of chlorotics. This aspect is very well explained by the results furnished by the analysis of the blood which was made by M. Favre.

1,000 parts of this blood gave of

| | |
|---|---|
| Fibrine | 4·420 |
| Globules | 44·400 |
| Solid materials of the serum | 76·554 |
| Water | 874·626 |
| | 1,000·000 |

This blood, in its composition, resembled that of chlorotics, both in the great diminution of its globules and the large proportion of water that it contained. And yet the patient to whom it belonged had certainly presented other symptoms than those of a simple chlorosis. The petechiæ and the echymoses with which the skin was covered are never met with in this disease; we may even say that nothing is more rare than to observe hemorrhage in chlorotics. As to the fibrine, far from being less abundant than in healthy blood, it was, on the contrary, raised above its physiological mean. This result is totally different from that which was furnished me by the analysis of the blood of another scorbutic, (of which the observation is published in the *Traité d'Hématologie Médicale*,) in which I found the globules in almost their normal proportions, and the fibrine, on the contrary, diminished. I have also found very little fibrine (about ·001) in the blood of a patient laboring under purpura hemorrhagica.

The fact which I have reported, and which is confirmative of the cases recently communicated to the academy by Messrs. Becquerel and Rodier, demonstrates that the symptoms which ordinarily characterize scurvy, may be produced without necessarily being accompanied by a diminution of the fibrine of the blood. It is not now in this diminution that we must place the proximate cause of scurvy, nor is it any more by it that we may hope to explain many of the symptoms of this disease, and in particular the numerous hemorrhages which constantly coincide with and characterize it.

In this connection, and in many others, perhaps, we may compare scurvy with typhoid fever. In the latter, the fibrine of the blood is in fact very frequently brought below its normal figure, but this is not essential to the existence of the disease. Observation authorizes us merely to lay down the principle, that in typhoid fever the fibrine diminishes in proportion as the dynamic form of the disease becomes more distinct. This is what also equally occurs in the eruptive fevers, in such a manner that in these various cases the diminution of the fibrine should not be considered as one of the necessary elements of the disease, but only as one of the possible and more or less frequent effects of the cause itself which has created it, and which exercises its influence equally upon the vital forces, which it tends to depress; upon the nervous system, where it gives rise to profound perturbation; and upon the blood, in which it tends to effect an alteration of composition, the reverse of that which occurs in the inflammatory state. The same thing appears to me to transpire in scurvy. Like typhoid fever, scurvy may be developed without the blood having previously degenerated in its fibrine. Consequently, in scurvy as in typhoid fever, the diminution of the fibrine of the blood will be neither a constant nor a necessary alteration; it will be but an effect—a result of anterior morbid modifications—a result which is produced more or less frequently according to the gravity, duration, etc. of the disease. Is it to this diminution of the fibrine of the blood that we should attribute the hemorrhages of scurvy? No doubt that these hemorrhages, which, like those of typhus, multiply and repeat themselves upon a great number of points of the economy, do show themselves especially in the cases where the blood is itself become poorer in fibrine than in the physiological condition.

But these two facts seem to me to have only the simple connection of coincidence. Both, without doubt, are most often produced by the intervention of a common cause; both are the manifestation of the action of this cause. But one of these facts, that of the diminution of the fibrine, does not appear to me to engender the other. The aberration that I have signalized, does it not in fact show us a case of scurvy where remarkable hemorrhages were produced, while the blood had not become poorer in its fibrine? Is it, moreover, very easy to comprehend theoretically how a diminution of quantity occurring in the fibrine of the blood, may induce a flow of this liquid from its vessels? Will it diminish the diameter of the globules, and may they traverse more easily the coats of the vessels? Shall it be admitted that this passage can be favored by an antecedent hyperemia, when after violent distensions of the vessels which are produced in phlegmasial congestion, this effect never occurs, and the globules never under such circumstances escape from their vessels, except in case of rupture of their parietes?

This opinion, revived in our time—one which I myself sustained—which made the hemorrhages of scurvy, like those of the grave fevers, depend upon the state of dissolution of the blood, was especially current at an epoch when it was supposed, that the dissolution of the blood was due to the destruction of its globules. But this destruction, held to exist by Huxham and so many others, is a pure hypothesis. Repeatedly in cases where the blood presented different degrees of this state of dissolution, so often described and so variously interpreted, I have examined this liquid under the microscope, and I always found the globules with their ordinary appearance, and perfectly intact. I have also often examined, in similar circumstances, the blood of hemorrhages, and I have invariably found the globules possessed of their physiological qualities. This being the case, I repeat it, one cannot understand how the simple fact of the diminution of the fibrine could render the escape of the globules from the circulatory channels more easy. From this it must not be concluded that, although there has been a very frequent and remarkable coincidence between the diminution of the fibrine of the blood and the production of hemorrhages, the first of these is the cause of the second, and that they may not exist independently of each other.

In the scorbutic who furnished me these reflections, not only had the blood not lost its fibrine, as in some of the facts cited by Messrs. Becquerel and Rodier, but was elevated in this respect above its physiological standard. In accounting for this, it must not be forgotten that at the moment when vene-

NB: Page nos 393-394 omitted in error. Text is complete.

section was practiced, symptoms of acute disease of the respiratory apparatus, with strong febrile action, were observed in our patient. It is to this circumstance that I believe myself able to attribute the existence of almost four and a half parts of fibrine in a thousand of his blood. Besides, a few days after the bleeding was practiced, and apparently by its influence, all febrile movements disappeared; and when, at a later period, we were able to examine the organ, we found no trace of this inflammatory action. While the blood at least two weeks before death had lost none of its fibrine, the spleen was very soft and reduced to a pulp; so that here is a circumstance where an alteration which is more commonly attendant on a state of dissolution of the blood, has shown itself without it. The lungs, which were violently congested, and filled here and there with apoplectic nuclei, presented a striking contrast, by their brown color, to the extreme paleness of the liver and kidneys.

To sum up: This fact proves that a well marked scurvy, already at an advanced stage, can exist without the blood presenting that state of dissolution which is commonly looked upon as attendant upon this disease, and without there being any diminution of the fibrine of the blood. Further observations will serve, perhaps, to account for this exceptional, though not unprecedented fact. From the fact that it is in opposition to the generally admitted opinion, and that it calls for a new examination, I have deemed it important to call the attention of pathologists to it.

Paris, July, 1847.

